# A Retrospective Analysis of Randomized Controlled Trials on Traumatic Brain Injury: Evaluation of CONSORT Item Adherence

**DOI:** 10.3390/brainsci11111504

**Published:** 2021-11-13

**Authors:** Meltem Elcivan, Ana Kowark, Mark Coburn, Hussam Aldin Hamou, Benedikt Kremer, Hans Clusmann, Anke Höllig

**Affiliations:** 1 Department of Neurosurgery, University Hospital RWTH Aachen, Pauwelsstr. 30, D-52074 Aachen, Germany; meltem.elcivan@rwth-aachen.de (M.E.); hhamou@ukaachen.de (H.A.H.); bkremer@ukaachen.de (B.K.); hclusmann@ukaachen.de (H.C.); 2 Department of Anaesthesiology, University Hospital RWTH Aachen, Pauwelsstr. 30, D-52074 Aachen, Germany; akowark@ukaachen.de; 3 Department of Anaesthesiology and Intensive Care Medicine, University Hospital Bonn, Venusberg-Campus 1, D-53127 Bonn, Germany; mark.coburn@ukbonn.de

**Keywords:** TBI, randomized controlled trial, CONSORT criteria, methodology

## Abstract

Traumatic brain injury (TBI) contributes to death and disability, resulting in an enormous individual and socio-economic challenges. Despite huge efforts, there are still controversies on treatment strategies and early outcome estimation. We evaluate current randomized controlled trials (RCTs) on TBI according to their fulfillment of the CONSORT (Consolidated Statement of Reporting Trials) statement’s criteria as a marker of transparency and the quality of study planning and realization. A PubMed search for RCTs on TBI (January 2014–December 2019) was carried out. After screening of the abstracts (*n* = 1.926), the suitable full text manuscripts (*n* = 72) were assessed for the fulfillment of the CONSORT criteria. The mean ratio of consort statement fulfillment was 59% (±13%), 31% of the included studies (*n* = 22) complied with less than 50% of the CONSORT criteria. Citation frequency was moderately related to ratio of CONSORT item fulfillment (*r* = 0.4877; *p* < 0.0001) and citation frequency per year (*r* = 0.5249; *p* < 0.0001). The ratio of CONSORT criteria fulfillment was associated with the impact factor of the publishing journal (*r* = 0.6428; *p* < 0.0001). Essential data for study interpretation, such as sample size determination (item 7a), participant flow (item 13a) as well as losses and exclusions (item 13b), were only reported in 53%, 60% and 63%, respectively. Reporting and methodological aspects in RCTs on TBI still may be improved. Thus, the interpretation of study results may be hampered due to methodological weaknesses.

## 1. Introduction

Traumatic brain injury (TBI) worldwide is one of the leading causes for disability. Data for 2016 reveal an age-standardized incidence of 369 (331–412) per 100,000 population for TBI globally [1]. Further, survivors suffer from disabilities [2] and have an increased risk for the occurrence of stroke, psychiatric diseases, dementia and other neurodegenerative disorders [3,4,5].

Randomized controlled trials (RCTs) are crucial for clinical decision-making and the creation of clinical guidelines. A transparent, complete and unbiased reporting on methods, conduct and results is essential to allowing a well-considered evaluation of the data. To increase the transparency of reporting, the Standardized Reporting of Trials (SORT) statement was first published in 1994 [3]. The statement was updated and merged with the Asilomar Guideline (by the Asilomar Working Group on Recommendations for Reporting of Clinical Trials in the Biomedical Literature; [4]) in 1996 (as the Consolidated Standards of Reporting Trials (CONSORT) Statement). Further refreshment took place in 2010 [3,5,6]. It is all the more important that the methodological and reporting issues are taken into account as TBI-specific aspects (as the heterogeneity of TBI [7,8]) may also influence the conduct of studies.

In 2015, Lu and colleagues provided an overview of CONSORT criteria compliance of RCTs on TBI (from 1976 to September 2013) and showed that several methodological aspects have improved over time [9]. However, the compliance of some fundamental items remained low and most studies seemed to be underpowered [9].

In order to update the available data, we analyzed the RCTs on TBI (January 2014–December 2019) with respect to their CONSORT criteria adherence and complemented the findings with a correlation of the citation frequencies and the impact factors of the publishing journals.

## 2. Materials and Methods

**Figure 1:**
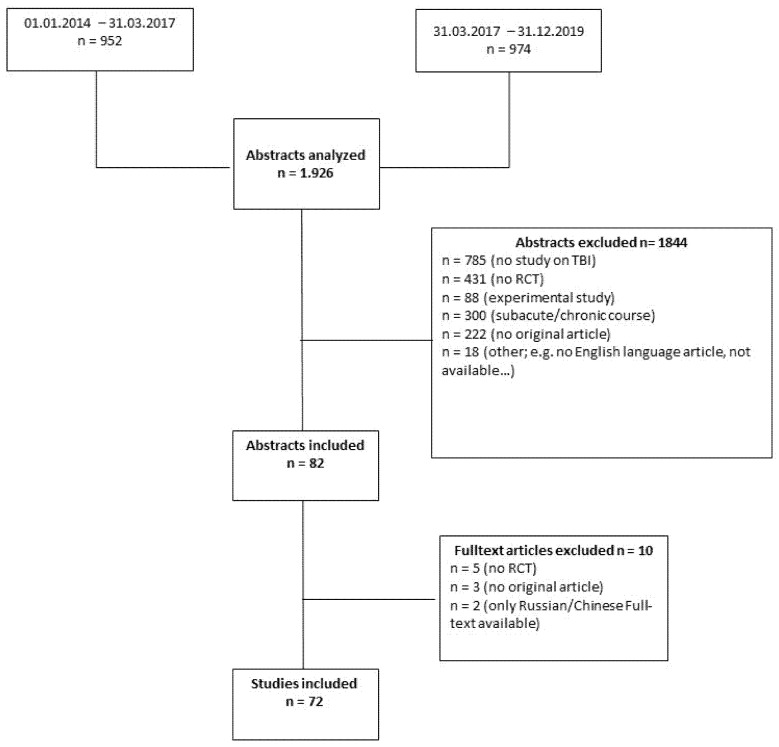
Flowchart of the studies included.

We performed a retrospective analysis of RCTs on TBI (published from January 2014 to December 2019) and reported the data according to the STROBE statement [10]. A PubMed search was carried out using the following search terms: [(traumatic brain injury) OR TBI OR (brain trauma) OR (head injury)] AND [(randomized controlled) OR randomized OR RCT]. Two authors (ME and AH) screened all abstracts for inclusion (applying the following inclusion criteria: randomized controlled clinical trial on patients with TBI within the acute treatment period). A Flowchart displays the excluded studies and the corresponding reasons for exclusion (see Flowchart; Figure 1). The included studies were evaluated according to their CONSORT criteria adherence by the first author (ME) [6]. A datasheet with precise descriptions of each item a priori was established in order to minimize subjective interpretation. Discrepancies regarding the study allocation were discussed with a second author (AH). Furthermore, 10% of all included RCTs were randomly double-checked by a second author (AH). Additionally, in the case of uncertainties about the assignment according to the CONSORT criteria, the specific item was discussed with a second author (AH). The journals’ websites were checked for supplemental material, which was included if available. Every item was classified as “fulfilled” (f), “not fulfilled” (nf) or “not applicable” (na). The classification “na” was used for items which are not mandatory, for example, item 7b, “When applicable, explanation of any interim analyses and stopping guidelines”.

According to the year the manuscript was published, the citation frequency and the impact factor of the journal were assessed using the Web of Science (Clarivate Analytics). The citation frequency per year was calculated to eliminate the bias derived from being available for a longer period of time. Some studies (specification see results section) were not available on the Web of Science. These studies were analyzed according to the CONSORT criteria adherence but correlation analyses with impact factor and citation frequency were not possible.

### Statistical Methods

For each item of the CONSORT criteria, the percentage of adherence was calculated. Additionally, summary statistics were assessed and graphically presented. Impact factor, citation frequency and citation frequency per year were correlated with CONSORT criteria fulfillment. As non-linear relationships were observed, we calculated the Spearman’s rank-order correlation (computing the coefficient r) and reported the corresponding *p*-value. For better visualization (due to non-linear relationship), logarithmic axes were used. Therefore, the graphical representation citation frequencies with a value of zero were set to 0.001. All our statistical analyses were performed using GraphPad Prism 9.1 (GraphPad Prism Software Inc., La Jolla, CA, USA). A p-value of 0.05 was regarded statistically significant.

## 3. Results

The PubMed search (January 2014–December 2019) resulted in 1926 abstracts (see Flowchart, Figure 1). A total of 1844 abstracts were excluded due to the following reasons: no study on TBI (*n* = 785), no RCT (*n* = 431), laboratory study (*n* = 88), delayed intervention (e.g., study on rehabilitation techniques; *n* = 300), no original article (*n* = 222), other reasons (*n* = 18).During the further analysis of the 82 studies left, five studies had to be excluded, as the full-text revealed that the studies did not meet the criteria of RCTs; two more articles had to be excluded as the full-texts were only available in Russian/Chinese; three articles were identified as review articles. Therefore, a total of 72 articles [11,12,13,14,15,16,17,18,19,20,21,22,23,24,25,26,27,28,29,30,31,32,33,34,35,36,37,38,39,40,41,42,43,44,45,46,47,48,49,50,51,52,53,54,55,56,57,58,59,60,61,62,63,64,65,66,67,68,69,70,71,72,73,74,75,76,77,78,79,80,81,82] were finally analyzed (see Flowchart, Figure 1). For four studies, no impact factor of the publishing journal was listed [16,38,39,40,76,77]. Further, 11 articles were not available through the Web of Science database search at all [12,15,33,41,51,55,56,66,74,79,81]. Details on the studies, including specific interventions, number of patients and outcome measures, are attached as a Supplemental Data File.

**Figure 2:**
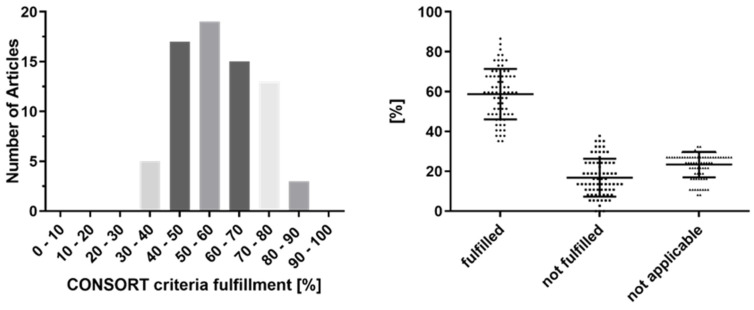
CONSORT criteria fulfillment: (**left**) numbers of articles according to their CONSORT criteria fulfillment; (**right**) distribution of CONSORT criteria fulfillment.

A mean ratio of fulfillment of 59% (±13%) was observed among the entire 37 CONSORT items. A total of 21 studies (29%) complied with less than 50% of the items (minimum: 35%). The highest fulfillment rate was observed with 87%. Of note, there are a few items, which do not necessarily have to be fulfilled (e.g., item 3b “Changes to trial design” or item 11b “Similarity of interventions: If relevant, description of the similarity of interventions”). The distribution of the CONSORT criteria fulfillment is shown in Figure 2.

The fulfillment of some items is essential in order to allow an appropriate assessment of the relevance of the results. Fundamental items, such as the determination of sample size (item 7a), participant flow (item 13a) and the indication of losses and exclusions (item 13b), were only fulfilled in about 60% of the studies (53%, 60% and 63%). The specific fulfillment ratios for each item are presented in Table 1.

**Figure 3:**
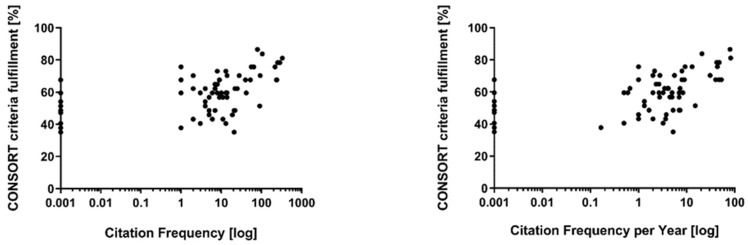
Correlation of citation frequency and CONSORT criteria fulfillment (**left**), citation frequency per year and CONSORT criteria fulfillment (**right**).

**Figure 4:**
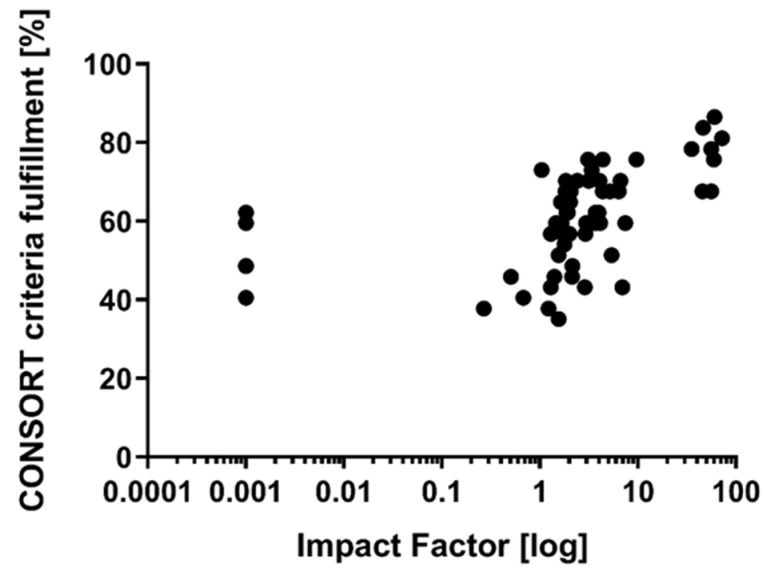
Correlation of impact factor of the publishing journal and CONSORT criteria fulfillment.

For six articles, no impact factor was available (although listed in the Web of Science), eleven articles were not listed at all. The mean impact factor of the articles available (*n* = 55) was 10.5 (±18.8; minimum: 0.269; maximum: 72.4). Citation frequency and citation frequency per year were correlated with the impact factor of the publishing journal (*r* = 0.5763 and *r* = 0.6526, both *p* < 0.0001). Both citation frequency and citation frequency per year, were moderately associated with CONSORT criteria fulfillment ratios (*r* = 0.4877 and *r* = 0.5249; both *p* < 0.0001) (see Figure 3). Higher impact factors of the publishing journal (assessed at the time of publication) were related to higher ratios of CONSORT criteria fulfillment (*r* = 0.6428; *p* < 0.0001) (see Figure 4).

## 4. Discussion

On average, the CONSORT criteria fulfillment ratios of studies on TBI are 59%. Essential items for the understanding of the study concept and conduct (e.g., sample size determination and participant flow) show low adherence. The citation frequency and impact factor of the publishing journals are related with the ratio of CONSORT criteria fulfillment.

In 2015, Lu and colleagues published an analysis of RCTs on TBI with regards to CONSORT criteria adherence comparing the timespans from 1976 to 2001, from 2002 to 2010 and from 2011 to 2013 [9]. They observed an improvement of reporting quality over time, but also a low overall quality and particularly small sample sizes indicating underpowered studies. Unfortunately, an in-depth comparison of the data with our results is not possible as results were not provided for every CONSORT item (but only the methodological ones).

Our results are similar to a previously published analysis of RCTs on intracranial hemorrhage (ICH) [83], but here we report a higher mean fulfillment ratio (average: 59% vs. 51%). This may be due to the different timeframe of inclusion (ICH study: January 2013–March 2017). Matching the current data with two earlier studies evaluating CONSORT criteria fulfillment of neurosurgical RCTs [84,85] the quality of reporting has improved slightly. In 2011, Kiehna and colleagues compared the CONSORT criteria adherence of RCTs published in four specific neurosurgical journals with those published in three leading general medicine journals (each published in 2006–2007) [84]. The adherence scores of the neurosurgical trials were far lower than those of the general medicine RCTs (mean score of 26.4 vs. 41 out of 44). Mansouri and colleagues analyzed neurosurgical RCTs from 2000 to 2014 using specific search terms [85]. Specific substantial items, such as sample size determination, were reported in only 20–34.2% (dependent on the sub-specialty of the study). On the one hand, only 50% of our analyzed studies reported information on sample size determination, which is an improvement compared to the results of Mansouri and colleagues. On the other hand, the description of sample size determination is an essential item for the interpretation of the study quality. Thus, a ratio of 50% is still disappointing.

Similar results were reported by Horton and colleagues, who particularly focused on the outcome measures in RCTs on TBI, and found heterogeneity in the use of outcomes and a variable quality of reporting [86]. The consequence of this lack of transparency is a possible misinterpretation of study results, which in turn may have consequences for clinical decision-making or the development of guidelines. Thus, adherence to the proper guidelines is not just about filling in the dots, but a necessity to reduce bias and to allow a complete and extensive understanding of the study. Further, it has to be emphasized that the mere fulfillment ratios do not take into account the importance of each item. Studies have to undergo an in-depth examination, as some items are essential for readers, reviewers and publishers in order to understand the intention and the concept of the study.

There is a vast amount of data documenting insufficient reporting in the health research literature regardless of the subspecialty and the ranking of the publishing journal [87,88,89]. Insufficient reporting is neither limited to neurosurgical topics nor is it a problem of RCTs. In fact, it is an important approach to increasing the sensitivity of the people involved (researchers, reviewer, publisher and readers) and make the problem public, as it is done by initiatives such as the EQUATOR network [90]. Awareness for data transparency and complete reporting (of positive and negative results) is needed [90,91]. Outcomes have to be defined properly, particularly when evidence exists that outcome measures are one problematic aspect of study conceptualization, which applies for TBI studies [7,8,86,92].

In 2015, Lu and colleagues have already mentioned that several journals publishing studies on neurotrauma endorse the CONSORT guidelines [9]. Assessing high-impact journals, the endorsement of the CONSORT statement increased substantially over time [93]. Thus, our observed correlation of the impact factor of the publishing journal and CONSORT criteria fulfillment may be due to the growing interest in methodology and a consecutive increase in the journals´ endorsements.

Our results are subjected to several limitations: Some of the CONSORT items allow a certain scope of interpretation. Therefore, the judgement may be biased by subjective weighting (only 10% of the data were cross-checked). We reported fulfillment of the items but did not prioritize specific items according to their significance. Some of the items do not apply to all studies (e.g., item 3b “Important changes to methods after trial commencement (such as eligibility criteria), with reasons”). We have not subcategorized these items. Further, only one database (PubMed) was searched. Lastly, the non-pharmacological extension of the CONSORT statement has not been considered (as published in 2017 and not yet available for the studies published from 2014–2017) [94].

## 5. Conclusions

In conclusion, we present data on the CONSORT criteria of studies on TBI (published from January 2014 to December 2019). The CONSORT criteria adherence ratios are low (59%). Bearing in mind that translational approaches in TBI research have largely failed, scientific approaches, methodology and reporting have to be reviewed. Adherence to certain quality standards should be requested by the journals. Importantly, these mandatory checklists should not only be a part of the submission process but also a transparent disclosure of the methodological approach and conduct of the study available for every reader. Further, the importance of methodological issues and the capability for critical analysis should be emphasized during the training of young academics.

## Figures and Tables

**Table 1:** Specific fulfillment ratios.

Item	Item Description		Items Fulfilled *n*/(%)
**Title and abstract**			
	1a	Identification as a randomised trial in the title.	***n*** ** = 42; 58.3%**
	1b	Structured summary of trial design, methods, results, and conclusions.	***n*** ** = 70; 98.6%**
**Introduction**			
Background and objectives	2a	Scientific background and explanation of rationale.	***n*** ** = 72; 100%**
	2b	Specific objectives or hypotheses.	***n*** ** = 68; 94.4%**
**Methods**			
Trial design	3a	Description of trial design (such as parallel, factorial) including allocation ratio.	***n*** ** = 70; 97.2%**
	3b	Important changes to methods after trial commencement (such as eligibility criteria), with reasons.	***n*** ** = 3; 4.2%**
Participants	4a	Eligibility criteria for participants.	***n*** ** = 72; 100%**
	4b	Settings and locations where the data were collected.	***n*** ** = 67; 93.1%**
Interventions	5	The interventions for each group with sufficient details to allow replication, including how and when they were actually administered.	***n*** ** = 71; 98.6%**
Outcomes	6a	Completely defined pre-specified primary and secondary outcome measures, including how and when they were assessed.	***n*** ** = 70; 97.2%**
	6b	Any changes to trial outcomes after the trial commenced, with reasons.	***n*** ** = 2; 2.8%**
Sample size	7a	How sample size was determined.	***n*** ** = 38; 52.8%**
	7b	When applicable, explanation of any interim analyses and stopping guidelines.	***n*** ** = 8; 11.1%**
Randomisation:			
Sequence generation	8a	Method used to generate the random allocation sequence.	***n*** ** = 59; 81.9%**
	8b	Type of randomisation; details of any restriction (such as blocking and block size).	***n*** ** = 35; 48.6%**
Allocation concealment mechanism	9	Mechanism used to implement the random allocation sequence (such as sequentially numbered containers), describing any steps taken to conceal the sequence until interventions were assigned.	***n*** ** = 30; 41.7%**
Implementation	10	Who generated the random allocation sequence, who enrolled participants, and who assigned participants to interventions.	***n*** ** = 10; 13.9%**
Blinding	11a	If done, who was blinded after assignment to interventions (for example, participants, care providers, those assessing outcomes) and how.	***n*** ** = 44; 61.1%**
	11b	If relevant, description of the similarity of interventions.	***n*** ** = 3; 4.2%**
Statistical methods	12a	Statistical methods used to compare groups for primary and secondary outcomes.	***n*** ** = 71; 98.6%**
	12b	Methods for additional analyses, such as subgroup analyses and adjusted analyses.	***n*** ** = 11; 15.3%**
**Results**			
Participant flow (a diagram is strongly recommended)	13a	For each group, the numbers of participants who were randomly assigned, received intended treatment, and were analysed for the primary outcome.	***n*** ** = 43; 59.7%**
	13b	For each group, losses and exclusions after randomisation, together with reasons.	***n*** ** = 45; 62.5%**
Recruitment	14a	Dates defining the periods of recruitment and follow-up.	***n*** ** = 60; 83.3%**
	14b	Why the trial ended or was stopped.	***n*** ** = 7; 9.7%**
Baseline data	15	A table showing baseline demographic and clinical characteristics for each group.	***n*** ** = 68; 94.4%**
	16	For each group, number of participants (denominator) included in each analysis and whether the analysis was by original assigned groups.	***n*** ** = 61; 84.7%**
Outcomes and estimation	17a	For each primary and secondary outcome, results for each group, and the estimated effect size and its precision (such as 95% confidence interval).	***n*** ** = 70; 97.2%**
	17b	For binary outcomes, presentation of both absolute and relative effect sizes is recommended.	***n*** ** = 2; 2.8%**
Ancillary analyses	18	Results of any other analyses performed, including subgroup analyses and adjusted analyses, distinguishing pre-specified from exploratory.	***n*** ** = 16; 22.2%**
Harms	19	All important harms or unintended effects in each group (for specific guidance see CONSORT for harms).	***n*** ** = 40; 55.6%**
**Discussion**			
Limitations	20	Trial limitations, addressing sources of potential bias, imprecision, and, if relevant, multiplicity of analyses.	***n*** ** = 57; 79.2%**
Generalisability	21	Generalisability (external validity, applicability) of the trial findings.	***n*** ** = 14; 19.4%**
Interpretation	22	Interpretation consistent with results, balancing benefits and harms, and considering other relevant evidence.	***n*** ** = 72; 100%**
**Other information**			
Registration	23	Registration number and name of trial registry.	***n*** ** = 40; 55.6%**
Protocol	24	Where the full trial protocol can be accessed, if available.	***n*** ** = 12; 16.7%**
Funding	25	Sources of funding and other support (such as supply of drugs), role of funders.	***n*** ** = 39; 54.2%**
		**mean**	**58.7%**

## Data Availability

Further data is available on request from the corresponding author.

## Supplementary Materials

The following are available online at https://www.mdpi.com/article/10.3390/brainsci11111504/s1, Table S1: overview of the studies analyzed.
