# Supplementary material for: A Retrospective Analysis of Randomized Controlled Trials on Traumatic Brain Injury: Evaluation of CONSORT Item Adherence
Source: Brain Sci. 2021 Nov 13;11(11):1504. doi: 10.3390/brainsci11111504 (PMC8615648; doi:10.3390/brainsci11111504)
Supplement: Supplementary file 1 [file brainsci-11-01504-s001.zip › brainsci-1390861-supplementary.pdf]

Table S1. overview of the studies analyzed.

Name of Study	Journal – Year of Publication	Interventions	Primary Outcome	Secondary Outcome
**20**: Evaluation of the Effect of Glibenclamide in Patients With Diffuse Axonal Injury Due to Moderate to Severe Head Trauma	Trauma Monthly – 11/2016	1. Intervention group (n=20): 1.25 mg Glibenclamide<br>2. Control group (n=20): not mentioned	GCS and GOS at discharge	Not particularly defined.
**33**: Early Enteral Combined with Parenteral Nutrition Treatment for Severe Traumatic Brain Injury: Effects on Immune Function, Nutritional Status and Outcomes	Chinese Medical Sciences Journal – 12/2016	1. EN: Enteral Nutrition Group (n=40)<br>2. PN: Parenteral Nutrition Group (n=40)<br>3. EN + PN: Enteral and Parenteral Nutrition Group (n=40)	LOS in NICU (length of stay in Neurological intensive care unit)	Number of Patients receiving AMV (assisted mechanical ventilation), length of AMV, death rate
**93**: Sympathetic activity and early mobilization in patients in intensive and intermediate care with severe brain injuries: a preliminary prospective randomized study	BMC Neurology – 09/2016	3 different protocols of mobilization:<br>1. Group 1 (n=10): Standard protocol<br>2. Group 2 (n=10): MOTOmed-letto® protocol<br>3. Group 3 (n=10): Erigo® protocol	Systolic blood pressure (SBP), diastolic blood pressure (DBP), heart rate (HR), respiratory rate (RR)	Plasma catecholamines, metanephrines and blood pressure before, during and after mobilization
**96**: Trial of Decompressive Craniectomy for Traumatic Intracranial Hypertension	New England Journal of Medicine – 09/2016	1. Intervention group (n=202): Decompressive Craniectomy<br>2. Control group (n=196): Barbiturate infusion	GOS-E at 6 months after randomization, mortality rate	Rates of vegetative state, lower severe disability, upper severe disability, lower moderate disability, upper moderate disability, lower good recovery, upper good recovery
**167**: Exogenous lactate infusion improved neurocognitive function of patients with mild traumatic brain injury	Asian Journal of Neurosurgery –05/2016	1. Intervention group (n=30): Hyperosmolar sodium lactate (HSL)<br>2. Control group (n=30): Hyperosmolar sodium chloride (HSS)	Mini-Mental State Examination (MMSE) score at 24h, 30 and 90d post-surgery	Hemodynamic and respiratory parameters: mean arterial pressure, heart rate, oxygen saturation
**298**: Comparing the Antiemetic Effects of Ondansetron and Metoclopramide in Patients with Minor Head Trauma	Emergency – 2015	1. Group A (n=60): Metoclopramide 10mg/2ml, slow injection<br>2. Group B (n=60): Ondansetron 4mg/2ml, slow injection	Mean nausea severity according to VAS in the twentieth minute post drug administration	Not particularly defined.

**314**: Hypothermia for Intracranial Hypertension after Traumatic Brain Injury	New England Journal of Medicine – 12/2015	1. Intervention group (n=195): Stage 2 treatments were added only if hypothermia failed to control intracranial pressure<br>2. Control group (n=192): Stage 2 treatments (e.g. osmotherapy) added as needed to control intracranial pressure<br>In both groups, stage 3 treatments (barbiturates and decompressive craniectomy) were used if all stage 2 treatments failed to control intracranial pressure	GOS-E at 6 months after injury	6-month mortality, lack of intracranial-pressure control, incidence of pneumonia, during days 1 through 7 after randomization, length of ICU-stay, grade on the modified Oxford Handicap Scale (MOHS)
**321**: A Prospective Randomised Study of Brain Tissue Oxygen Pressure-Guided Management in Moderate and Severe Traumatic Brain Injury Patients	Biomed Research International – 08/2015	1. Intervention group (n=23): PbtO2-guided management, PbtO2 > 20 mmHg<br>2. Control group (n=27): ICP-guided therapy, ICP < 20 mmHg and CPP > 60 mmHg	Survival rate at 3 and 6 months after injury, GOS-E from 1 to 6 months after injury	Not particularly defined.
**344**: Surgical Trial In Traumatic intraCerebral Haemorrhage (STITCH): a randomised controlled trial of Early Surgery compared with Initial Conservative Treatment	Health Technology Assesment – 09/2015	1. Intervention group (n=83): Early Surgery within 12 hours<br>2. Control group (n=87): Initial Conservative treatment	Death or severe disability on the GOS, which was recorded using a self-completed structured questionnaire based on the 8-point GOS	Rankin, EQ-5D-3L, Mortality, Survival, Major Adverse Events (death, pulmonary embolism or deep-vein thrombosis, infection, rebleeding), QALYs, total health-care costs, social costs
**372**: Neuroprotective Effects of Erythropoetin in Patients with Severe Closed Brain Injury	Turkish Neurosurgery – 07/2015	1. Intervention group (n=24): EPO administration<br>2. Control group (n=18): no intervention	GOS-E scores at 6 months	Mortality at 6 months, proportion of surviving patients with unfavorable neurological outcome at 6 months,
**406**: Intensive versus conventional glucose control in critically ill patients with traumatic brain injury: long-term follow-up of a subgroup of patients from the NICE-SUGAR study	Intensive Care Medicine – 06/2015	1. Intervention group (n=203): Intensive blood glucose control<br>2. Control group (n=188): Conventional blood glucose control	GOS-E (includes mortality) at 24 months	The incidence of moderate and severe hypoglycemia, duration of ICU stay and hospital stay, the number of patients treated and the duration of treatment with mechanical ventilation and renal replacement therapy

**477**: The Head Injury Retrieval Trial (HIRT): a single-centre randomised controlled trial of physician prehospital management of severe blunt head injury compared with management by paramedics only"	Emergency medicine journal – 03/2015	1. Intervention group (n=197): Standard treatment plus treatment by a physician arriving by helicopter<br>2. Control group (n=178): Standard treatment (ground paramedic response)	30-day mortality and GOS at 6 months	Duration of stay in hospital and the intensive care unit, 30-day mortality and rates of hypotension, hypoxia at first contact compared with hospital arrival
**489**: Early Surgery versus Initial Conservative Treatment in Patients with Traumatic Intracerebral Hemorrhage: The first randomized trial	Journal of Neurotrauma –05/2015	1. Intervention group (n=83): Early surgery, hematoma evacuation within 12 h of randomization<br>2. Control group (n=87): Conservative treatment group (n=87): subsequent evacuation allowed if deemed necessary randomization within 48 h of TBI	6-month GOS dichotomized into favorable and unfavorable outcome; dead, vegetative, and severe disability were coded as unfavorable and moderate disability and good recovery as favorable	Mortality, time to death, extended Glasgow Outcome Scale (GOSE), Rankin, European Quality of Life Five Dimension Scale (EQ-5D) at 6 months
**541**: Benefits of Strict Rest After Acute Concussion: A Randomized Controlled Trial	Pediatrics – 02/2015	1. Usual care group (n=50): 1–2 days rest, followed by stepwise return to activity<br>2. Strict rest group (n=49): Maintain 5 days of strict rest at home (specifically, no school, work, or physical activity)	The Three-Day Activity Diary: Physical activity, mental activity, PCSS (19 item)	Not particularly defined.
**596**: Prolonged Mild Therapeutic Hypothermia versus Fever Control with Tight Hemodynamic Monitoring and Slow Rewarming in Patients with Severe Traumatic Brain Injury: A Randomized Controlled Trial	Journal of Neurotrauma – 07/2014	1. Intervention group (n=98): Therapeutic hypothermia: 32–34°C, patients cooled as soon as possible for ≥72 h and rewarmed at a rate of <1°C/day<br>2. Control group (n=50): Fever control: 35.5–37°C	GCS at 6 months	Severe disability (SD), persistent vegetative state (PVS), or death (D), moderate disability (MD) or good recovery (GR)
**12**: Effects of citicoline on level of consciousness, serum level of fetuin-A and matrix Gla-protein (MGP) in trauma patients with diffuse	Ulusal travma ve acil cerrahi dergisi –11/2014	1. Intervention group: Citicoline: 500 mg every 6 hours<br>2. Control group: not mentioned<br>n=58 in total	GCS	Mean serum levels of Fetuin-A and of Matrix Gla Protein (MGP)

axonal injury (DAI) and GCS≤8				
**24**: A Clinical Trial of Progesterone for Severe Traumatic Brain Injury	New England Journal of Medicine – 12/2015	1. Intervention group (n=591): Progesterone<br>2. Control group (n=588): Placebo	GOS at 6 months after the injury	GOS score at 3 months, mortality at 1 month and 6 months, GOS-E
**25**: Very Early Administration of Progesterone for Acute Traumatic Brain Injury	New England Journal of Medicine – 12/2014	1. Intervention group (n=442): Progesterone<br>2. Control group (n=440): Placebo	GOS-E at 6 months after injury	Mortality, the Disability Rating Scale score, the rates of nine prespecified adverse events
**105**: Early Pressure Dressing for the Prevention of Subdural Effusion Secondary to Decompressive Craniectomy in Patients With Severe Traumatic Brain Injury	Journal of Craniofacial Surgery – 09/2014	1. Intervention group (n=82): Early pressure dressing with an elastic bandage<br>2. Control group (n=87): only general wrapping with a light bandaging	GOS at 6 months after injury	Death, vegetative state, severe disability, moderate disability, good recovery
**106**: Hydrocortisone and fludrocortisone for prevention of hospital-acquired pneumonia in patients with severe traumatic brain injury (Corti-TC): a double-blind, multicenter phase 3, randomised placebo-controlled-trial	Lancet Respiratory Medicine – 07/2014	1. Intervention group (n=168): Hydrocortisone (intravenous continuous infusion of 200 mg per day for 7 days starting on day 1, 100 mg on days 8 and 9, and 50 mg on day 10)<br>2. Control group (n=168): Placebo	Hazard ratio for HAP (hospital-acquired pneumonia) at day 28	Cases of HAP per patient, duration of mechanical ventilation before HAP, cases of HAP (n), clicinal criteria for diagnosis of HAP, Respiratory tract samples, other infections, Antibiotic-free days at day 28, mean duration of mechanical ventilation, days without mechanical ventilation at day 28, mean duration of intensive care, ICU-free days at day 28
**108**: Effect of Erythropoietin and Transfusion Threshold on Neurological Recovery After Traumatic Brain Injury A Randomized Clinical Trial	Jama-Journal of the American medical association – 07/2014	1. Intervention group (n=102): Erythropoietin<br>2. Control group (n=98): Placebo	GOS at 6 months post injury	Mortality for patients assigned to erythropoietin or placebo
**167**: Nitric oxide synthase inhibition with the antipterin VAS203 improves outcome in moderate and severe traumatic brain injury: a placebo-controlled	Journal of Neurotrauma – 07/2014	1. Open Cohort 1 (n=8): three 12-h-intravenous (iv) infusions of VAS203<br>2. Cohort 2 & 3 (n=24): either VAS203 or placebo	GOS-E at 6 months after injury	Metabolites of VAS203 in cerebral microdialysates

randomized Phase Iia trial (NOSTRA)				
**202**: Hyperglycemia and antibody titres against heat shock protein 27 in traumatic brain injury patients on parenteral nutrition	Iranian Journal of basic medical sciences – 02/2014	1. Intervention group (n=13): Intensive insuline treatment IIT<br>2. Control group (n=13): Twice daily blood glucose control TDGC	Anti HSP27 titre over the study period	Not particularly defined.
**229**: The efficacy of cyclosporine-A on diffuse axonal injury after traumatic brain injury	Adv Biomed Res - 01/2014	1. Intervention group (n=50): 5 mg/kg/24 h via 250 ml dextrose water (DW) 5% solution (DW 5%) during the first 8 h after trauma<br>2. Control group (n=50): only DW 5% in the same course	GOS-E 3 and 6 months after trauma	Mini-mental state examination (MMSE) at 3 and 6 months after trauma
**233**: Hypertonic Saline as a Therapy for Pediatric Concussive Pain A Randomized Controlled Trial of Symptom Treatment in the Emergency Department	Pediatric emergency care – 03/2014	1. Intervention group (n=23): Hypertonic saline (HTS)<br>2. Control group (n=21): Normal saline (NS)	Change in self-reported pain following fluid administration	Change in pain, postconcussive symptoms within 2 to 3 days of fluid administration
**239**: Recombinant human interleukin-1 receptor antagonist in severe traumatic brain injury: a phase II randomized control trial	Journal of Cerebral blood flow and metabolism – 02/2014	1. Intervention group (n=10): Recombinant human IL1ra (rhIL1ra, anakinra) at a dose of 100 mg subcutaneously once a day for 5 days<br>2. Control group (n=10): no intervention, monitored in an identical manner with sampling time points identical to those in the intervention group	Resultant cerebral cytokine profile: extracellular concentrations of IL1ra and 41 cytokines and chemokines	Not particularly defined.
**197**: An equiosmolar study on early intracranial physiology and long term outcome in severe traumatic brain injury comparing mannitol and hypertonic saline	Journal of Clinical Neuroscience – 05/2016	1. Intervention group (n=20): Mannitol<br>2. Control group (n=18): HTS	GOS at 6 months	Not particularly defined.

**2**: Intensive insulin therapy for preventing postoperative infection in patients with traumatic brain injury: A randomized controlled trial.	Medicine – 03/2017	1. Intervention group (n=44) ITT: Intensive insulin therapy<br>2. Control group (n=44) CIT: Conventional insulin therapy	GOS, infections (pneumonia, sepsis, urinary, wound infections) according to the National Nosocomial Infection Surveillance System	Duration of ICU stay and mortality rate at 6 months after injury
**11**: Early Administration of Selenium in Patients with Acute Traumatic Brain Injury: A Randomized Double-blinded Controlled Trial.	Indian Journal of critical care Medicine – 02/2017	1. Intervention group (n=57): Selenium 500 µg intravenously at 100 ml normal saline for 30 min and then 500 µg at 100 ml normal saline during 24 h continuously for 14 days in addition to standard care<br>2. Control group (n=56): Routine standard treatment alone	GOS-E at 2 months after injury	Change in APACHE III score on the 15th day, daily changes in FOUR score and SOFA score within 15 days of first interventions, side effects of selenium, length of Intensive Care Unit (ICU) stay, and length of hospital stay
**17**: The effect of tranexamic acid in traumatic brain injury: A randomized controlled trial.	Chinese Journal of Traumatology – 02/2017	1. Intervention group (n=40): Tranexamic acid<br>2. Control group (n=40): Placebo	The extent of ICH growth at 48 h after admission	Not particularly defined.
**144**: Effects of Normobaric Hyperoxia in Traumatic Brain Injury: A Randomized Controlled Clinical Trial.	Trauma Monthly – 02/2016	1. Intervention group (n=34): 80% Oxygen by mechanical ventilator in the first 6 hours after the traumatic accident<br>2. Control goup (n=34): 50% Oxygen by mechanical ventilator in the first 6 hours after the traumatic accident	GOS at 6 months after injury	Length of Stay, Barthel Index, and Modified Rankin Scale
**145**: Influence of two anesthetic techniques on blood sugar level in head injury patients: A comparative study.	Anesthesia Essays and Researches – 05/2016	1. Intervention group (n=30): Induction with sevoflurane and then had O2 + air + sevoflurane for maintenance with controlled ventilation<br>2. Control group (n=30): Induction with i.v. propofol and then had O2 + air + propofol for maintenance with controlled ventilation	GCS	Not particularly defined.

**220**: What are the progesterone-induced changes of the outcome and the serum markers of injury, oxidant activity and inflammation in diffuse axonal injury patients?	International Immunopharmacology – 03/2016	1. Intervention group (n=24): Progresterone<br>2. Control group (n=24): not mentioned	GOS-E + FIM scores 6 months after injury	Markers of inflammation [interleukin-1β (IL-1β), IL-6, transforming growth factor-β1 (TGF-β1)], injury (brain protein of S-100B), and oxidant activity [malondialdehyde (MDA)
**312**: Erythropoietin in traumatic brain injury (EPO-TBI): a double-blind randomised controlled trial.	Lancet – 12/2015	1. Intervention group (n=308): Erythropoietin alfa 40.000 IE as a subcutaneous injection<br>2. Control group (n=298): 0.9% sodium chloride as a subcutaneous injection	GOS-E at 6 months after injury	Mortality, proximal deep venous thrombosis detected by ultrasound, occurrence of a composite thrombotic outcome
**476**: Hypothermia for Traumatic Brain Injury in Children-A Phase II Randomized Controlled Trial.	Critical Care Medicine – 07/2015	1. Intervention group (n=28): Therapeutic hypothermia to a temperature of 32–33°C for 72 hours followed by slow rewarming at a rate compatible with maintaining intracranial pressure and cerebral perfusion pressure<br>2. Control group (n=27): Strict normothermia to a temperature of 36–37°C for 72 hours	Pediatric cerebral performance category (PCPC), eligibility and recruitment rates, protocol violation, major adverse events	ICP and CPP during the first 5 days and treatments required, duration of mechanical ventilation, PICU, hospital lengths of stay, adverse events including infectious complications, bleeding, pancreatitis, ARDS, arrhythmias
**10**: Neuromuscular electrical stimulation in critically ill traumatic brain injury patients attenuates muscle atrophy, neurophysiological disorders, and weakness: a randomized controlled trial	Journal of Intensive Care –12/2019	1. Intervention group (n=30): Daily Neuromuscular electrical stimulation (NMES) for 14 days in the lower limb muscles<br>2. Control group (n=30): Only conventional physiotherapy	Effect of NMES over the muscle architecture, the presence of NED, and the evoked peak force	Plasma level of cytokines and metalloproteinases, mechanical ventilation time, length of stay in the ICU, length of hospitalization
**38**: The effects of peppermint gel on prevention of pressure injury in hospitalized patients with head trauma in neurosurgical ICU: A double-	Complementary Therapies in Medicine – 12/2019	1. Intervention group (n=75): Peppermintgel three times a day up to 14 days during the skin care as a layer on the skin areas exposed to the risk of pressure injuries	Incidence of pressure injuries stage I via National Pressure Ulcer Advisory Panel	Not particularly defined.

blind randomized controlled trial		2. Control group (n=75): Placebo gel		
**81:** Effects of tranexamic acid on death, disability, vascular occlusive events and other morbidities in patients with acute traumatic brain injury (CRASH-3): a randomised, placebo-controlled trial	Lancet – 11/2019	1. Intervention group (n=4649): A loading dose of 1 g of tranexamic acid infused over 10 min, started immediately after randomisation, followed by an intravenous infusion of 1 g over 8 h<br>2. Control group (n=4553): Placebo	Head injury-related death in hospital within 28 days of injury assessed by the responsible clinician	Early head injury-related death (within 24 h after injury), all-cause and cause-specific mortality, disability, vascular occlusive events (myocardial infarction, stroke, deep vein thrombosis, and pulmonary embolism), seizures, complications, neurosurgery, days in intensive care unit, adverse events within 28 days of randomization
**111:** Low-Dose, Early Fresh Frozen Plasma Transfusion Therapy After Severe Trauma Brain Injury: A Clinical, Prospective, Randomized, Controlled Study	World Neurosurgery – 12/2019	1. Intervention group (n=28): Treatment with fresh frozen plasma (5 mL/kg body weight; LEFT group)<br>1) Control group (n=35): Normal saline (5 mL/kg body weight; NO LEFT group)	GOS at 6 months after injury	Not particularly defined.
**112**: Safety and efficacy of Cerebrolysin in acute brain injury and neurorecovery: CAPTAIN I-a randomized, placebo-controlled, double-blind, Asian-Pacific trial	Neurological Sciences – 12/2019	1. Intervention group (n=22): 50 mL of Cerebrolysin daily for 10 days<br>2. Control group (n=24): Physiological saline solution daily for 10 days	A multidimensional ensemble of 14 outcome scales pooled to be analyzed by means of the multivariate, correlation-sensitive Wei-Lachin procedure	Not particularly defined.
**119:** Beta-adrenergic blockade for attenuation of catecholamine surge after traumatic brain injury: a randomized pilot trial	Trauma Surgery & Acute Care Open – 08/2019	1. Intervention group (n=13): Propranolol treatment<br>2. Control group (n=13): Placebo	In-hospital mortality	The interaction between beta-blockade and catecholamines by measuring urinary catecholamines
**120:** Effects of dexmedetomidine vs sufentanil during percutaneous tracheostomy	Medicine – 08/2019	1. D1 (n = 62): DEX infusion at $0.5 \mu g \cdot kg^{-1}$ for 10 minutes, then adjusted to $0.2–0.7 \mu g \cdot kg^{-1} \cdot hour^{-1}$)	Hemodynamic variables: MAP and HR	Paroxysmal sympathetic hyperactivity (PSH) score, anesthesia onset time, duration of anesthesia and surgery, total cumulative dose of

for traumatic brain injury patients: A prospective randomized controlled trial		2. D2 (n=68): DEX infusion at 1 μg·kg^{-1} for 10 minutes, then adjusted to 0.2–0.7 μg·kg^{-1}·hour^{-1})<br>3. S (n=66): sufentanil infusion 0.3 μg·kg^{-1} for 10 minutes, then adjusted to 0.2–0.4 μg·kg^{-1}·hour^{-1})		DEX/sufentanil, time to first dose of rescue propofol and fentanyl, total doses of rescue propofol and fentanyl, number of intraoperative patient movements, surgeon satisfaction score, and adverse events (such as bradycardia, tachycardia, hypotension, hypertension, and respiratory depression)
**177:** Efficacy of Simultaneous Administration of Nimodipine, Progesterone, and Magnesium Sulfate in Patients with Severe Traumatic Brain Injury: A Randomized Controlled Trial	Bulletin of Emergency And Trauma –04/2019	1. Intervention group (n=45): Intravenous nimodipine 60 mg, intramuscular progesterone 1 mg/kg and magnesium sulfate 5 grams stat followed by 2.5 grams<br>2. Control group (n=45): Placebo	GCS, SjvO2: jugular venous oxygen saturation, GOS	Not particularly defined.
**211:** A randomized controlled trial on the efficacy, safety, and pharmacokinetics of metformin in severe traumatic brain injury	Journal of Neurology – 08/2019	1. Intervention group (n=15): 1 g metformin two times a day at 12-h intervals for five consecutive days through a nasogastric tube<br>2. Control group (n=15): Usual management only	5-day post-trauma serum concentration profile of S100B	Longitudinal changes of serum GFAP and NLR
**249:** Comparison of equiosmolar dose of hyperosmolar agents in reducing intracranial pressure-a randomized control study in pediatric traumatic brain injury	Childs Nervous System – 03/2019	1. Intervention group (n=16): Treatment with 20% mannitol<br>2. Control group (n=14): 3% saline as 2.5 ml/kg bolus for episodes of intracranial pressure above cutoff value for age	The mean reduction in intracranial pressure and GOS at 6 months after injury	Not particularly defined.
**250:** Transfusion requirements after head trauma: a randomized feasibility controlled trial	Critical Care – 03/2019	1. Liberal group (n=23): Transfusion if hemoglobin concentration < 9 g/dL<br>2. Restrictive group (n=24): Transfusion if hemoglobin concentration < 7 g/dL	The mean difference in hemoglobin between groups	Transfusion requirements, intracranial pressure management, cerebral hemodynamics, length of stay, mortality and 6-month neurological outcome

**273:** Enoxaparin in the treatment of severe traumatic brain injury: A randomized clinical trial	Surgical Neurology International – 01/2019	1. Intervention group (n=26): 0.5 mg/kg enoxaparin subcutaneously every 6 h in six total doses<br>2. Control group (n=27): Placebo	The radiological appearance of a new ICH or an increase in the size of the previous ICH	The clinical outcome of the patient at discharge, favorable outcome versus poor outcome, or death
**357:** Comparison of Phenytoin versus Levetiracetam in Early Seizure Prophylaxis after Traumatic Brain Injury, at a Tertiary Care Hospital in Karachi, Pakistan	Asian Journal of Neurosurgery –10/2018	1. Group A (n=70): Intravenous phenytoin monotherapy<br>2. Group B (n=70): Intravenous levetiracetam monotherapy	Follow up-GCS	Seizure activity
**377:** Effect of Early Sustained Prophylactic Hypothermia on Neurologic Outcomes Among Patients With Severe Traumatic Brain Injury The POLAR Randomized Clinical Trial	Jama-Journal of The American Medical Assocaition – 12/2018	1. Intervention group (n=266): Hypothermia (33°C-35°C)<br>2. Control group (n=245): Normothermia (37°C)	GOS-E 5-8 [scale range, 1-8] obtained by blinded assessors or Severity-adjusted relative risk for favorable outcome (using IMPACT-TBI) 6 months after injury	Death at 6th month, infections, pneumonia, bacteremia, bleeding
**423:** Therapeutic hypothermia to reduce intracranial pressure after traumatic brain injury: the Eurotherm3235 RCT	Health Technology Assessment – 08/2018	1. Intervention group: Hypothermia (32–35 °C)<br>2. Control group: Standard care alone<br><br>n=387 in total	GOS-E at 6 months after the injury	Intracranial pressure control, incidence of pneumonia across both groups, length of stay in the ICU and hospital, Modified Oxford Handicap Scale score at 1 month, correlation between the predicted outcome using the MOHS score at hospital discharge and the predicted outcome, health economics
**295:** Comparison of Effects of Manual and Mechanical Airway Clearance Techniques on Intracranial Pressure in Patients With Severe Traumatic Brain Injury on a Ventilator: Randomized, Crossover Trial	Physical Therapy – 04/2019	1. Group A (n=23): Treatment with a manual chest percussion technique<br>2. Group B (n=23): Treatment with a mechanical chest wall vibrator<br><br>(Each treatment for 10 minutes alternately, separated by an interval of 4 hours)	ICP	Cerebral perfusion pressure, heart rate, mean arterial pressure, and arterial blood gas parameters
**701:** Effect of Tranexamic Acid on Prevention of Hemorrhagic Mass Growth in	World Neurosurgery – 01/2018	1. Intervention group (n=78): Tranexamic acid (TXA),1 gram per 1000 mL of normal saline for 8 hours	Growth of the hemorrhagic mass	Need of surgery, death, unfavorable outcome at discharge (GOS),

Patients with Traumatic Brain Injury		2. Control group (n=78): Placebo, 0.9% normal saline used in the same order		unfavorable outcome at 3 months after discharge (GOS)
**713:** Brain Oxygen Optimization in Severe Traumatic Brain Injury Phase-II: A Phase II Randomized Trial	Critical Care Medicie – 11/2017	1. Intervention group (n=57): Intracranial pressure plus brain tissue oxygenation monitoring (PbtO2) 2. Control group (n=62): Intracranial pressure monitoring alone	GOS-E at 6 months after discharge	Not particularly defined.
**727:** The Profile of MMP-9, MMP-9 mRNA Expression, -1562 C/T Polymorphism and Outcome in High-risk Traumatic Brain Injury: The Effect of Therapeutic Mild Hypothermia	Neurologia Medico-Chirurgica – 08/2017	1. Intervention group (n=10): Mild hypothermia therapy 2. Control group (n=10): Therapy without mild hypothermia	Full Outline of UnResponsiveness (FOUR) score and Glasgow Coma Scale (GCS)	The MMP-9 level, MMP-9 mRNA expression and -1562 C/T polymorphism using ELISA, -restriction fragment length polymorphism (PCR-RFLP)
**787:** Effect of Memantine on Serum Levels of Neuron-Specific Enolase and on the Glasgow Coma Scale in Patients With Moderate Traumatic Brain Injury	Journal of Clinical Pharmacology – 01/2018	1. Intervention group (n=22): Enteral memantine 30 mg twice daily for 7 days 2. Control group (n=19): Standard TBI management	GCS, mean neuron-specific enolase (NSE) serum level	Not particularly defined.
**798**: Effects of atorvastatin on brain contusion volume and functional outcome of patients with moderate and severe traumatic brain injury; a randomized double-blind placebo-controlled clinical trial	Journal of Clicinal Neuroscience – 11/2017	1. Intervention group (n=21): Daily 20mg atorvastatin for 10days 2. Control group (n=23): Placebo in the same dosage	Modified Rankin scale (MRS), GOS-E and Disability rating Scale (DRS) at 3 months after injury	Not particularly defined.
**820:** Effects of immunonutrition on biomarkers in traumatic brain injury patients in Malaysia: a prospective randomized controlled trial	BMC Anesthesiology – 06/2017	1. Group A (n=18): Immunonutrition, Neomune (is enriched with arginine, glutamine and omega-3 fatty acid) 2. Group B (n=18): Enteral feed, Fresubin® HP energy	Levels of biomarkers (IL-6, glutathione, CRP, total protein and albumin)	Not particularly defined.

**827:** The effects of family-centered affective stimulation on brain-injured comatose patients' level of consciousness: A randomized controlled trial	Internatinal Journal of Nursing Studies – 09/2017	1. Experimenatal group (n=37): Affective stimulation intervention by family members<br>2. Placebo group (n=36): Sensory stimulation program by a fixed trained person (not familiar with the patients)<br>3. Control group (n=37): Sensory stimulation routinely provided to all patients	GCS, Coma Recovery Scale-Revised	Not particularly defined.
**865:** Impact of Zinc Supplementation on the Clinical Outcomes of Patients with Severe Head Trauma: A Double-Blind Randomized Clinical Trial	Journal of Dietary Supplements – 2018	1. Intervention group (n=50): Zinc solution (528 mg zinc sulfate and distilled water)<br>2. Control group (n=50): Placebo (20 mL of distilled water); each therapy enterally for 15 days	Mortality rate and GOS at discharge	Not particularly defined.
**871:** Optimization of brain metabolism using metabolic-targeted therapeutic hypothermia can reduce mortality from traumatic brain injury	Journal of Trauma and Acute Care Surgery – 08/2017	1. Metabolic-targeted hypothermia treatment (MTHT) group (n=52): 50% to 60% rest metabolic ratio (RMR) as the hypothermia therapy<br>2. Body temperature-targeted hypothermia treatment (BTHT) control group (n=50): Hypothermia therapy of 32°C to 35°C body temperature	Mortality	Disturbances of metabolic networks via H nuclear magnetic resonance technology
**878:** The effect of hypertonic saline and mannitol on coagulation in moderate traumatic brain injury patients	American Journal of Emergency Medicine – 10/2017	1. Group A (n=43): 20% Mannitol (MT)<br>2. Group B (n=40): 3% Hypertonic saline (HTS)	ROTEM parameters included CT (clotting time), CFT (clot formation time), maximum clot firmness (MCF) measured by MCF (EXTEM and INTEM), MCF (FIBTEM) and standard coagulation tests	Not particularly defined.

**931:** Mild induced hypothermia for patients with severe traumatic brain injury after decompressive craniectomy	Journal of Critical Care – 06/2017	1. Intervention group (n=30): Hypothermia (32°C-35°C)<br>2. Control group (n=30): Standard care	GOS at 6 months after injury	Mortality rate
**921:** Effects of Oral Glibenclamide on Brain Contusion Volume and Functional Outcome of Patients with Moderate and Severe Traumatic Brain Injuries: A Randomized Double-Blind Placebo-Controlled Clinical Trial	World Neurosurgery – 05/2017	1. Intevention group (n=29): Glibenclamide<br>2. Control group (n=23): Placebo	GOS; Modified Rankin Scale (MRS), Disability Rating Scale (DRS); each at 3 months	Not particularly defined.
**480:** Benefits of the tranexamic acid in head trauma with no extracranial bleeding: a prospective follow-up of 180 patients	European Journal of Trauma and Emergency Surgery – 08/2019	1. Intervention group (n=96): Therapy with Tranexamic Acid (TXA)<br>2. Control group (n=84): No therapy with Tranexamic Acid (TXA)	Benefits of TXA in reducing the needing of surgery or transfusion and the mortality rate up to 28 days after trauma	The safety of TXA in TBI including pulmonary embolism or deep vein thrombosis and disability using GOS at the 28th day after trauma
**515:** The effects of amantadine on traumatic brain injury outcome: a double-blind, randomized, controlled, clinical trial	Brain injury – 2018	1. Intervention group (n=20): Amantadine as 100mg twice a day<br>2. Control group (n=22): Placebo	Mini-Mental State Examination (MMSE), GOS, Disability Rating Scale, and Karnofsky Performance Scale (KPS)	Not particularly defined.
**538:** Comparison of effect of dexmedetomidine and lidocaine on intracranial and systemic hemodynamic response to chest physiotherapy and tracheal suctioning in patients with severe traumatic brain injury	Journal of Anesthesia – 08/2018	1. Group I (n=30): Dexmedetomidine 0.5 mcg/kg (IV)<br>2. Group II (n=30): Lidocaine 2 mg/kg (IV)	Heart rate, MAP, peak airway pressure (AWP_{peak}), SpO_2 and ICP	Not particularly defined.
**563:** Role of neomycin polymyxin sulfate solution bladder wash for prevention of catheter associated urinary tract infection in traumatic brain injury patient admitted	International Journal of Critical Illness & Injury Science – 01/2018	1. Intervention group (n=50): Bladder wash with Neomycin and Polymyxin Sulphate solution<br>2. Control group (n=50): Bladder wash with Normal saline bladder wash	Incidence of Catheter - associated urinary tract infection (CAUTI)	Not particularly defined.

to Intensive Care Unit: A prospective randomized				
**590:** Recombinant human erythropoietin for treating severe traumatic brain injury	Medicine – 01/2018	1. Intervention group (n=60): Recombinant human erythropoietin (RHE)<br>2. Control group (n=): 0.9% Saline	GOS at dicharge, mortality	Any adverse events.
**616:** Balanced Fluid Versus Saline-Based Fluid in Post-operative Severe Traumatic Brain Injury Patients: Acid-Base and Electrolytes Assessment	Malaysian Journal of Medical Sciences – 09/2017	1. Group B (n=33): Sterofundin ISO<br>2. Group S (n=33): Normal Saline 0,9%	Acid-base parameters and electrolytes analysed using repeated measures ANOVA.	Not particularly defined.
**617:** Target-controlled Infusion Propofol Versus Sevoflurane Anaesthesia for Emergency Traumatic Brain Surgery: Comparison of the Outcomes	Malaysian Journal of Medical Sciences – 09/2017	1. Group T (n=55): Anaesthesia with Propofol target plasma concentration of 3–6 μg/mL<br>2. Group (n=55): with minimum alveolar concentration (MAC) of Sevoflurane 1.0–1.5	GOS at discharge	Rate of successful extubation amon the survival, mortality rate
**621:** Cerebral metabolic effects of strict versus conventional glycaemic targets following severe traumatic brain injury	Critical Care Medicine – 01/2018	1. Intervention group: Strict (4–7 mmol/L; 72–126 mg/dl) glycaemic control<br>2. Control group: Conventional (<10 mmol/L; 180 mg/dl) glycaemic control<br>(Therapy in both groups (n=20) performed with microdialysis measurements hourly)	GOS at 6 months, APPACHE II, GCS, Intensive Care Unit (ICU), Injury Severity Score (ISS)	Not particularly defined.
**643:** To Compare the Effect of Two Different Doses of Dexmedetomidine on the Attenuation of Airway and Pressor Response during Tracheostomy Tube Change in Traumatic Brain Injury Patients	Anesthesia Essays and Researches –10/2017	1. Group A (n=30): Dexmedetomidine (DEX) 0.5 μg/kg<br>2. Group B (n=30): Dexmedetomidine (DEX) 1.0 μg/kg	The hemodynamic response (heart rate, mean arterial pressure) of tracheal stimulation and cough reflex scaling	Not particularly defined.

**680:** Randomized controlled study comparing 2 surgical techniques for decompressive craniectomy: with watertight duraplasty and without watertight duraplasty	Journal of Neurosurgery –10/2017	1. Intervention group (n=): Therapy with watertight duraplasty<br>2. Control group (n=): Therapy without watertight duraplasty	Incidence of surgical complications (CSF leak, wound infection, brain abscess, or subgaleal fluid collections)	Clinical outcome (analyzed using the Glasgow Outcome Scale [GOS]), surgical time, and hospital costs
